# Research progress on melatonin, 5-HT, and orexin in sleep disorders of children with autism spectrum disorder

**DOI:** 10.17305/bb.2024.11182

**Published:** 2024-11-08

**Authors:** Wenjun Ding, Yiran Xu, Wencong Ding, Qiongyan Tang, Bohao Zhang, Yangyang Yuan, Jian Jin

**Affiliations:** 1 Scientific Research Center, The Third Affiliated Hospital of Zhengzhou University, Zhengzhou, China; 2 Intervention Neurosurgery Department, The Third Affiliated Hospital of Zhengzhou University, Zhengzhou, China; 3 Neurology Department, The First Affiliated Hospital of Zhengzhou University, Zhengzhou, China

**Keywords:** Autism spectrum disorder, sleep disorders, melatonin, 5-HT, orexin

## Abstract

Sleep disorders are among the common comorbidities of autism spectrum disorder (ASD), which not only affect the daily life and learning ability of children but may also exacerbate other symptoms of ASD, seriously impacting the quality of life of children and their families. Given this, understanding the neurobiological mechanisms of sleep disorders in children with ASD has significant research value for developing effective intervention strategies. Melatonin, 5-hydroxytryptamine (5-HT), and orexin are key neurotransmitters that regulate the sleep–wake cycle. Through in-depth analysis of the biological functions and regulatory pathways of these neurotransmitters, new perspectives may be provided for personalized treatment of sleep disorders in children with ASD. This article reviews the research progress on melatonin, 5-HT, and orexin in sleep disorders among children with ASD, focusing on exploring the mechanisms of these key neurotransmitters in sleep disorders of children with ASD and how they affect the sleep–wake cycle, providing a theoretical basis for improving the sleep quality of children with ASD.

## Introduction

Autism spectrum disorder (ASD) is a group of neurodevelopmental disorders characterized by communication and social interaction difficulties, limited interests, and repetitive stereotyped behaviors [1]. As our understanding of ASD deepens, it has been found that children with ASD often have comorbidities across multiple systems, with sleep disorders being particularly common [2]. The clinical manifestations of sleep disorders in children with ASD are diverse, mainly including difficulty falling asleep, delayed sleep onset, light sleep, shortened sleep duration, daytime sleepiness, and insomnia [3]. Additionally, children with ASD may experience symptoms, such as difficulty breathing during sleep and sleep-related breathing pauses. Some studies also indicate that children with ASD may have sleep-related movement disorders, such as restless leg syndrome, periodic limb movements during sleep, periodic limb movement disorder, and rhythmic movement disorder [4, 5]. Restless leg syndrome can cause sleep onset issues, while periodic limb movements can lead to nighttime awakenings and excessive daytime sleepiness.

**Figure 1.**
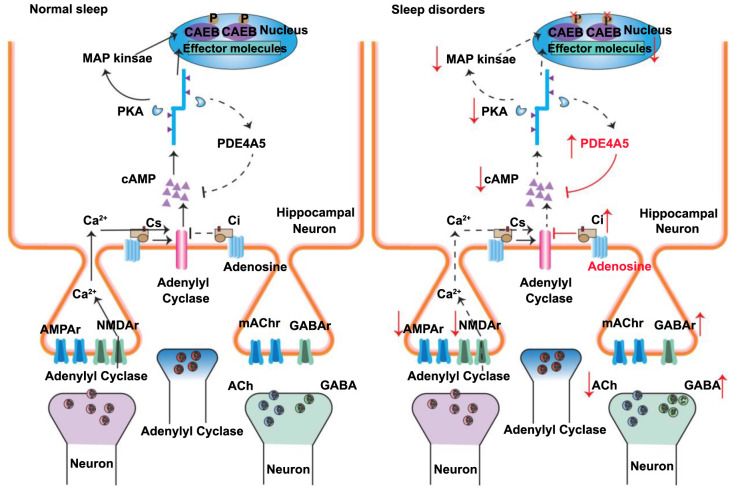
**Signal transmission in the hippocampus under different sleep states.** PKA: Protein kinase A; cAMP: Cyclic adenosine monophosphate; GABA: Gamma-aminobutyric acid.

**Figure 2.**
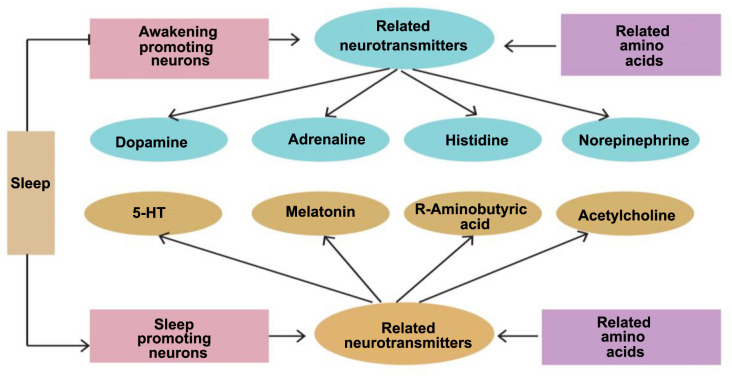
**Regulation of neurotransmitters related to sleep.** 5-HT: 5-hydroxytryptamine.

Reynolds et al. [6] conducted a questionnaire survey with 522 parents of children with ASD aged 2–5 years, finding that 48.5% of the children had at least one sleep problem. Sleep disorders may disrupt normal signal transduction in the hippocampus (as shown in Figure 1 below), thus affecting daily life and learning abilities. Sleep disorders may also exacerbate other ASD symptoms, seriously impacting the quality of life of affected children and their families. Therefore, it is particularly urgent to conduct in-depth research on sleep disorders in children with ASD and explore their potential biological mechanisms. The pathogenesis of sleep disorders in children with ASD is currently not fully understood, but it may be related to abnormal regulation of certain key neurotransmitters in the brain (as shown in Figure 2). Common neurotransmitters involved in sleep regulation include melatonin, 5-hydroxytryptamine (5-HT), orexin, histamine, prostaglandins, dopamine, and acetylcholine. Melatonin and orexin are neurotransmitters that directly regulate the sleep–wake cycle; 5-HT contributes to emotional stability and wakefulness during the day and can be converted into melatonin at night to regulate sleep [7–9]. These three neurotransmitters play a central role in sleep regulation, making their roles in sleep disorders in children with ASD particularly noteworthy. However, the expression and function of these neurotransmitters in children with ASD have not been fully elucidated, especially concerning the interactions and balance among these neurotransmitters in maintaining normal sleep patterns. Therefore, studying the mechanisms of melatonin, 5-HT, and orexin in sleep disorders in children with ASD may not only help us better understand the sleep problems of children with ASD but also provide a scientific basis for developing targeted interventions. Regulating the levels and functions of these key neurotransmitters could improve sleep quality in children with ASD, thereby enhancing their overall quality of life. This study aims to summarize the research progress on these neurotransmitters in sleep disorders in children with ASD through a literature review, analyze how they affect sleep patterns in children with ASD, and provide a scientific basis for the clinical treatment and intervention of sleep disorders in children with ASD.

## The main mechanisms of sleep disorders in children with ASD

The pathogenesis of sleep disorders in children with ASD is currently unclear and may be related to various factors, including abnormal sleep regulation mechanisms, core symptoms, comorbidities, physical organic lesions, nutritional status, and medication.

### Neuropathological mechanisms

Sleep disorders in children with ASD may be related to neuropathological mechanisms, particularly dysfunction of the hypothalamic–pituitary–adrenal axis that regulates circadian rhythms [10]. This dysfunction may lead to abnormal metabolic pathways of related neurotransmitters, especially the metabolic pathway of melatonin [11]. In children with ASD, the electroencephalogram (EEG) during rapid eye movement (REM) sleep exhibits abnormalities, such as spindle-shaped EEG waves and EEG activity unique to premature infants and infants aged 3–8 months, while lacking the slow-wave activity and REM burst activity observed in typical children during REM sleep [12]. These abnormalities indicate that children with ASD have issues with the composition and maturation of sleep brainwaves. Additionally, children with ASD may experience increased muscle activity during the REM phase, which may lead to periodic limb movement syndrome and REM sleep behavior disorders, further suggesting abnormalities in factors related to sleep–wake regulation [13]. These factors may include monoamine pathways, neurotransmitters, synapses, and abnormal development of nervous system components [14].

Beyond abnormalities in sleep–wake regulation, gene mutations related to circadian rhythms are also an important mechanism of sleep disorders in children with ASD [15]. Genes known to regulate human circadian rhythm include the PERIOD gene family (such as PER1 and PER2), TIMELESS, NPAS2, and Cryptochromes (such as CRY1 and CRY2). Some gene mutations may simultaneously affect the development of the nervous system and sleep, such as mutations in the NRXN1 and SHANK3 genes, which may influence sleep in children with ASD by affecting synaptic health in the central nervous system (CNS) and peripheral nervous system (PNS) [16, 17].

### Core symptoms of ASD

The core symptoms of ASD mainly include social communication disorders and stereotyped, repetitive behaviors, interests, or activities. These symptoms affect the daily lives of affected children to varying degrees, including their sleep patterns. Firstly, social disorders may lead to difficulties in understanding social cues and routines in children with ASD, including sleep-related daily habits and nonverbal cues expected by parents [18]. For example, they may struggle to recognize or respond to signals from their parents indicating bedtime, such as dimming lights or reducing evening activities, making it difficult to transition smoothly to sleep.

Secondly, children with ASD often exhibit stereotyped and repetitive behaviors that may be out of sync with their internal physiological rhythms, leading to disruptions in the sleep–wake cycle [19]. They may have a strong dependence on certain daily activities or environmental factors, and changes to these factors may disrupt their sleep patterns. Additionally, children with ASD often show resistance to new experiences or changes, making it challenging to alter their sleep habits or adapt to new sleep environments [20]. They may have strict requirements for pre-bedtime routines, and any changes can lead to anxiety and sleep disturbances. Children with ASD may also experience difficulties in emotional regulation, which can result in heightened anxiety and excitement at night, making it hard to calm down and fall asleep [21]. They may need more time to relax and unwind from daytime activities, posing a barrier to sleep onset. Finally, communication barriers with parents may also affect the sleep of children with ASD. Due to challenges with language and nonverbal communication, they may struggle to express their feelings or needs, including issues related to sleep, making it difficult for parents to identify and address specific problems impacting their child’s sleep.

## The role of melatonin in the sleep of children with ASD

### Basic properties of melatonin

**Figure 3.**
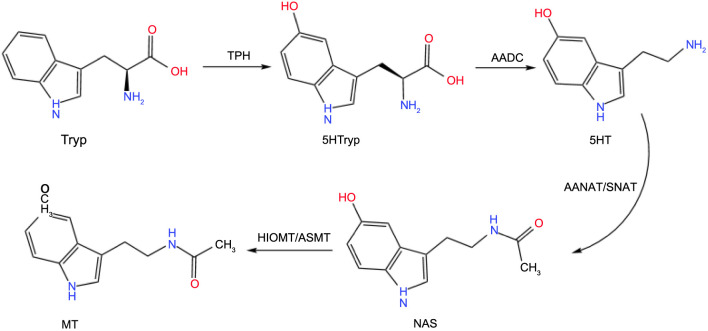
**Melatonin synthesis in mammals.** AANAT: Arylalkylamine N-acetyltransferase; SNAT: Serotonin N-acetyltransferase; HIOMT: Hydroxyindole-O-methyltransferase; 5-HT: 5-hydroxytryptamine; ASMT: N-acetyl-5-hydroxytryptamine-O-methyltransferase.

Melatonin is an endogenous neurohormone produced by the pineal gland in mammals, which regulates the sleep–wake cycle by providing photoperiod information to the body [22]. In mammals, melatonin biosynthesis mainly uses tryptophan as a substrate and involves four synthesis steps (as shown in Figure 3): The first step is the formation of 5-hydroxytryptophan (5-HTP) under the catalysis of tryptophan hydroxylase (TPH). In the second step, 5-HTP is catalyzed by L-aromatic amino acid decarboxylase (AAAD) to form serotonin. In the third step, serotonin is acetylated by serotonin N-acetyltransferase (SNAT) or arylalkylamine N-acetyltransferase (AANAT) to produce N-acetyl-5-hydroxytryptamine. Finally, N-acetylserotonin is methylated by N-acetyl-5-hydroxytryptamine-O-methyltransferase (ASMT) or hydroxyindole-O-methyltransferase (HIOMT) to synthesize melatonin [23]. Once synthesized, melatonin is rapidly released into the systemic circulation, reaching central and peripheral target tissues and exerting physiological functions [24]. While most tissues and organs in the body can secrete melatonin, only the pineal gland and retina exhibit circadian rhythms in melatonin synthesis. The circadian rhythm of melatonin is characterized by low levels during the day and high levels at night, peaking approximately 2 h before habitual sleep time, reaching a plateau during the night, and decreasing upon waking [25].

### The mechanism of melatonin in regulating sleep in children with ASD

Current studies [26–28] have found that children with ASD exhibit abnormal patterns of melatonin secretion, such as delayed melatonin peaks, decreased amplitude, and reduced nighttime secretion, which may lead to circadian rhythm sleep–wake disorders (CRSWDs) in children with ASD. Studies [28, 29] have reported that mutations in genes encoding melatonin-metabolizing enzymes (ASMT, AANAT, and CYP1A2) and melatonin receptor genes (MTNR1A, MTNR1B, and GPR50) in children with ASD may be the cause of abnormal melatonin metabolism. Mechanistically, melatonin’s role in the sleep of children with ASD involves complex biochemical processes, including the regulation of signaling pathways and cytokines. Melatonin acts through its specific membrane and nuclear receptors, which are expressed in multiple brain regions, including the suprachiasmatic nucleus (SCN), where the main biological clock resides [30]. One of the main signaling pathways of melatonin is through its G protein-coupled receptors MT1 and MT2, which, when activated, can inhibit the production of cyclic adenosine monophosphate (cAMP), thereby affecting various intracellular signaling molecules, such as protein kinase A (PKA) and extracellular signal-regulated kinases (ERK) [31, 32]. Changes in these signaling molecules can regulate neuronal excitability and affect transitions in sleep–wake cycles. On a cytokine level, melatonin has anti-inflammatory effects and can reduce the expression of pro-inflammatory cytokines, such as tumor necrosis factor-alpha (TNF-α) and interleukin-6 (IL-6), both of which play important roles in sleep regulation [33, 34]. The anti-inflammatory effects of melatonin help maintain stable sleep structures and reduce sleep disorders.

### Application of melatonin in the treatment of ASD sleep disorders

The treatment of ASD should primarily involve sleep hygiene and behavioral interventions. When these measures do not produce satisfactory results, pharmacotherapy may be considered. Commonly used medications include alpha agonists, antidepressants, and atypical antipsychotics. The sleep problems of children with ASD are often related to abnormal secretion and metabolism of melatonin, so exogenous melatonin supplementation therapy may be effective. So far, the best evidence for the use of melatonin in children is for insomnia caused by circadian rhythm sleep disorders, as it theoretically can shorten sleep onset latency and increase total sleep time [35, 36]. The International Pediatric Sleep Association (IPSA) recommends using sustained-release melatonin for children with ASD who struggle to maintain sleep, while short-acting melatonin is recommended for those who have trouble falling asleep [37]. Schroder et al. [38] found that exogenous melatonin supplementation not only improves sleep but also has a positive effect on externalized behavioral difficulties in children with ASD, significantly improving the quality of life for caregivers. Malow et al. [39] demonstrated that slow-release melatonin can improve the sleep of children with ASD without negatively impacting their growth and development.

## The association between 5-HT and sleep disorders in children with ASD

### Basic characteristics of 5-HT

5-HT is an important neurotransmitter and local hormone that plays diverse physiological roles in the human body. It is synthesized through the enzymatic reaction of tryptophan, first converted to 5-HTP and then further processed into 5-HT [40]. 5-HT is primarily involved in regulating emotions, sleep, appetite, and pain perception in the brain [41]. It functions by activating different receptor subtypes, which are widely distributed in the central and PNSs, including seven major families: 5-HT1 to 5-HT7 [42]. Signal transmission of 5-HT involves multiple intracellular pathways and can affect nerve transmission and cellular function. In terms of emotional regulation, changes in 5-HT levels are associated with various emotional disorders such as depression [43]. Popova et al. [44] noted in their research that depression, aggression, and suicidal behavior are associated with dysregulation of central 5-HT neurotransmission. In addition, 5-HT plays a central role in regulating the sleep cycle, particularly in promoting sleep and maintaining normal sleep structures [45]. Kang et al. [46] found that regulating the 5-HT pathway can improve fatigue in sleep-deprived mice. The synthesis and release of 5-HT are influenced by light and circadian rhythms, which in turn affect melatonin production and the sleep–wake cycle [47]. Therefore, 5-HT is not only crucial in the nervous system but is also indispensable in maintaining overall physiological balance.

### Mechanisms of 5-HT in regulating sleep in children with ASD

In terms of sleep regulation, 5-HT acts as a neurotransmitter that promotes wakefulness and slow-wave sleep, and its abnormal secretion can directly lead to disturbances in the sleep–wake cycle [48]. Currently, abnormal 5-HT metabolism in children with ASD has been documented, including increased serum levels, decreased 5-HT transporters, and polymorphism of the SLC6A4 gene encoding 5-HT transporters [49–51]. In children with ASD, dysfunction of the 5-HT system is closely associated with the occurrence of sleep disorders. 5-HT affects sleep structure and cycle through its receptors. For example, activation of 5-HT2A receptors is associated with promoting wakefulness, while 5-HT1A receptors are associated with promoting sleep [52, 53]. In children with ASD, changes in the sensitivity or expression levels of 5-HT receptors may lead to an imbalance in sleep–wake cycle regulation. Additionally, 5-HT may indirectly affect sleep by influencing physiological processes, such as thermoregulation, gastric emptying, and intestinal motility. On a molecular level, 5-HT synthesis is catalyzed by TPH, and TPH activity is influenced by gene polymorphisms [54]. Specific TPH gene variations are linked to sleep disorders in children with ASD, potentially by altering the synthesis and availability of 5-HT. The level of 5-hydroxyindoleacetic acid (5-HIAA), a metabolite of 5-HT, in cerebrospinal fluid is also associated with sleep quality in children with ASD [55]. 5-HT also participates indirectly in sleep regulation by affecting other neurotransmitter systems, such as melatonin and dopamine. For example, 5-HT influences melatonin secretion, which is a key hormone in regulating the biological clock and sleep cycle. Interactions between 5-HT and dopamine are also involved in maintaining wakefulness and initiating sleep. In children with ASD, abnormalities in the 5-HT system may be linked to specific behavioral characteristics, such as repetitive behavior and social disorders, which can further affect sleep patterns. For example, repetitive behavior may decrease nighttime wakefulness and sleep efficiency.

### Application of 5-HT in the treatment of sleep disorders in ASD

Research on the treatment of sleep disorders in children with ASD using 5-HT has shown some progress. Due to its central role in regulating emotions and sleep, the 5-HT system has become a potential target for treating ASD-related sleep problems. For instance, selective serotonin reuptake inhibitors (SSRIs) are a class of drugs that increase 5-HT concentration in the synaptic cleft [56]. In children with ASD, SSRIs may help alleviate emotional issues related to sleep disorders, such as anxiety and depression, thereby indirectly improving sleep quality [56]. Some atypical antidepressants, such as trazodone, have an antagonistic effect on 5-HT2A receptors and have been used to treat insomnia. These drugs may also have therapeutic potential for sleep disorders in children with ASD [57]. Additionally, regulating 5-HT through traditional Chinese medicine has been shown to effectively improve sleep quality [58]. It is important to note that when choosing 5-HT-related drugs for treatment in clinical practice, individualized evaluation of patients is necessary to minimize possible complications.

## The effect of orexin on sleep disorders in children with ASD

### Basic characteristics of orexin

Orexin, also known as hypothalamic secretin, is a group of neuropeptides produced in the hypothalamus that widely affect other areas of the brain. It includes two forms: orexin A and orexin B [59]. These neuropeptides exert their effects by binding to specific G protein-coupled receptors—orexin receptor 1 (OX1R) and orexin receptor 2 (OX2R)—whose distribution in the brain is closely related to physiological processes, such as the sleep–wake cycle, energy metabolism, feeding behavior, and stress response [60, 61]. The activity of orexins is mainly related to promoting wakefulness, reducing sleep, and enhancing feeding behavior [62]. In addition, the orexin system is involved in regulating emotions, cognitive function, and addictive behavior [63]. Abnormal activity of orexin neurons is directly related to various sleep disorders, especially hypersomnia [64]. Excessive secretion of orexins at night can lead to a heightened wakefulness system, making it difficult to transition smoothly to the sleep system, resulting in insomnia. If the activity of orexins at night can be inhibited or the binding between orexins and appetite hormone receptors blocked, it will inhibit the wakefulness-promoting effect of orexins, reduce awakening signals, increase drowsiness, and restore a normal sleep rhythm [65]. The discovery of orexins provides important clues for understanding the neurobiological mechanisms of sleep, wakefulness, and related diseases.

### Mechanisms of orexin in regulating sleep in children with ASD

In children with ASD, this neuropeptide may affect sleep patterns through specific neural pathways and genetic mechanisms. Specifically, orexin enhances the activity of arousal-promoting neurotransmitters, such as dopamine and norepinephrine in the brain, while inhibiting sleep-promoting neurotransmitters, such as gamma-aminobutyric acid (GABA) and serotonin (5-HT), thus affecting the sleep–wake cycle [66]. Orexin may also influence the circadian rhythm of the sleep–wake cycle by affecting the circadian function of the SCN [67]. Additionally, the orexin signaling pathway may alter sleep patterns by influencing the expression of genes related to the sleep–wake cycle, such as Clock and Bmal1 [68]. Orexin receptor antagonists (ORAs) help restore a normal sleep rhythm by blocking the binding of orexin to its receptors, reducing arousal signals, increasing drowsiness, and promoting sleep [69]. Orexin may also impact sleep and wakefulness states by influencing pathways related to neural plasticity, such as the expression of brain-derived neurotrophic factor (BDNF) [70]. Therefore, the mechanism of the orexin signaling pathway in sleep disorders in children with ASD is a complex process involving multiple aspects, such as neurotransmitter system regulation, interactions between brain regions, influence on the biological clock, gene expression regulation, and stress response modulation.

### Application of orexin in the treatment of ASD sleep disorders

In the past two decades, with the growing understanding of the orexin system’s role, particularly in regulating wakefulness, ORAs have shown significant potential as a new type of neurological or psychiatric drug for treating insomnia, making them a hot topic in drug research [71, 72]. Currently, three innovative ORA drugs—suvorexant, lemborexant, and daridorexant—have been approved by the U.S. FDA for treating adult patients with insomnia [73–75]. These three drugs are dual ORAs (DORAs) that can be taken orally. Although no ORA is currently available for treating sleep disorders in children with ASD, regulating orexin system activity may help improve sleep patterns in ASD patients by reducing excessive wakefulness or adjusting the sleep–wake cycle, as orexin mainly promotes wakefulness and inhibits sleep. Additionally, children with ASD often have other mental and neurodevelopmental disorders, such as anxiety, depression, or attention deficit hyperactivity disorder (ADHD). Appetite regulation may also positively impact these comorbid sleep problems.

## The mechanism of sleep disorders in ASD children under the interaction of multiple neurotransmitters

In children with ASD, the formation of sleep disorders results from the interaction and combined regulation of multiple neurotransmitters on sleep–wake cycles. Changes in the concentration of a single neurotransmitter cannot fully explain the pathophysiological mechanisms of these sleep disorders. There are increasing experimental studies on the mutual influence of various neurotransmitters on sleep disorders, as well as on the roles and pathogenic mechanisms of neurotransmitters and transmission pathways in sleep disorders. The increased secretion of melatonin at night typically marks the beginning of sleep, while the 5-HT system affects mood and wakefulness during the day through its various receptor subtypes. Additionally, orexin, through the activation of its receptors OX1R and OX2R, not only directly promotes wakefulness but may also inhibit melatonin secretion, affecting the onset of sleep. In children with ASD, the balance between these neurotransmitters may be disrupted. For example, changes in 5-HT levels may impact the synthesis and secretion of melatonin, leading to disturbances in the sleep–wake cycle [76]. Meanwhile, orexins may influence melatonin secretion by inhibiting β-epinephrine, making it difficult for children with ASD to enter deep sleep at night [77, 78]. This imbalance may also relate to changes in the sensitivity or expression levels of 5-HT receptors, which may vary in ASD children due to genetic and environmental factors.

The interaction between orexin and 5-HT also involves regulation of the stress response, which is particularly significant in children with ASD, as they may be more sensitive to daily stress. An enhanced stress response may further affect the release of orexins by activating the 5-HT system, contributing to sleep disorders [79]. Furthermore, the interaction of these neurotransmitters may affect other brain areas related to sleep regulation, such as the SCN. As the main brain region regulating biological rhythms, the SCN may be jointly influenced by changes in melatonin, 5-HT, and orexin levels [80]. Therefore, the mechanism of sleep disorders in children with ASD likely involves the interaction of these neurotransmitters at multiple levels, including direct neural regulation, impact on biological rhythms, and regulation of stress responses. Understanding the specific mechanisms of these interactions is crucial for developing targeted treatments, which may help improve the sleep quality of children with ASD by regulating the balance between these neurotransmitters. Future research needs to explore the role of these neurotransmitters in sleep disorders in children with ASD and how to restore their normal function through medication, behavioral therapy, or other interventions.

## Conclusion

In summary, the abnormal regulation of melatonin, 5-HT, and orexin—three key neurotransmitters—may be closely related to sleep disorders in children with ASD. Abnormal circadian secretion of melatonin may disrupt sleep–wake cycles. Dysfunction of the 5-HT system directly affects the regulation of emotions and sleep. Excessive secretion of orexin may hinder the smooth transition of the sleep system. Although there is a preliminary understanding of the mechanisms by which these neurotransmitters contribute to sleep disorders in children with ASD, the specific molecular mechanisms and signaling pathways are not yet fully elucidated. This is especially true regarding the interactions between these three key neurotransmitters and others, such as histamine, prostaglandins, dopamine, and acetylcholine, and their roles in sleep disorders in children with ASD. Future research should focus on the mechanisms of sleep disorders involving the interaction of multiple neurotransmitters and on ways to regulate these neurotransmitters through medication, behavioral therapy, or other interventions to restore normal sleep patterns. Additionally, developing personalized treatment strategies that consider the genetic and environmental differences among children with ASD will be key to improving treatment outcomes. As an emerging therapeutic approach, ORAs have shown potential in regulating the sleep–wake cycle, but their application in children with ASD requires further clinical research to validate their safety and efficacy. Ultimately, by comprehensively considering neurobiological, genetic, and environmental factors, we aim to provide more comprehensive and effective interventions for sleep disorders in children with ASD, thereby improving their quality of life.
